# A mixed methods inquiry into the validity of data

**DOI:** 10.1186/1751-0147-50-30

**Published:** 2008-07-22

**Authors:** Erling Kristensen, Dorte B Nielsen, Laila N Jensen, Mette Vaarst, Carsten Enevoldsen

**Affiliations:** 1 StrateKo Aps, Gartnervaenget 2, DK-8680 Ry, Denmark; 2 Department of Large Animal Sciences, Faculty of Life Sciences, University of Copenhagen, Grønnegaardsvej 2, DK-1870 Frederiksberg C, Copenhagen, Denmark; 3 Department of Animal Health, Welfare and Nutrition, Faculty of Agricultural Sciences, University of Aarhus, Research Centre Foulum, P.O. 50, DK-8830 Tjele, Denmark

## Abstract

**Background:**

Research in herd health management solely using a quantitative approach may present major challenges to the interpretation of the results, because the humans involved may have responded to their observations based on previous experiences and own beliefs. This challenge can be met through increased awareness and dialogue between researchers and farmers or other stakeholders about the background for data collection related to management and changes in management. By integrating quantitative and qualitative research methods in a mixed methods research approach, the researchers will improve their understanding of this potential bias of the observed data and farms, which will enable them to obtain more useful results of quantitative analyses.

**Case description:**

An example is used to illustrate the potentials of combining quantitative and qualitative approaches to herd health related data analyses. The example is based on two studies on bovine metritis. The first study was a quantitative observational study of risk factors for metritis in Danish dairy cows based on data from the Danish Cattle Database. The other study was a semi-structured interview study involving 20 practicing veterinarians with the aim to gain insight into veterinarians' decision making when collecting and processing data related to metritis.

**Discussion and Evaluation:**

The relations between risk factors and metritis in the first project supported the findings in several other quantitative observational studies; however, the herd incidence risk was highly skewed. There may be simple practical reasons for this, e.g. underreporting and differences in the veterinarians' decision making. Additionally, the interviews in the second project identified several problems with correctness and validity of data regarding the occurrence of metritis because of differences regarding case definitions and thresholds for treatments between veterinarians.

**Conclusion:**

Studies where associations between specific herd health management routines and disease outcome variables are drawn based purely on quantitative observational studies may benefit greatly by adding a qualitative perspective to the quantitative approach as illustrated and discussed in this article. The combined approach requires, besides skills and interdisciplinary collaboration, also openness, reflection and scepticism from the involved scientists, but the benefits may be extended to various contexts both in advisory service and science.

## Background

Herd Health Management (HHM) has emerged from veterinary sciences and related fields primarily as a response to the increasing herd sizes [1]. HHM is characterized by the use of knowledge encompassing numerous disciplines, especially epidemiology and veterinary clinical sciences, but also business and herd health economics, law, sociology, psychology and ethics as embedded in philosophy of life and involvement of the farmer [2]. Consequently, professionals working with HHM need to combine knowledge and skills related to cows, housing system, management strategies, human behaviour and all relevant interactions to understand the different stakeholders involved in a dairy setting. This is consistent with the view of Schwabe *et al*. [3]: "In a herd health type situation, field research should be virtually indistinguishable from practice", indicating that the HHM approach in practice is an analytical approach, which involves many different disciplines. Consequently, HHM research must combine diverse types and fields of knowledge and research.

HHM professionals aim at making a difference in the dairy setting in terms of a) improvement of production and b) improvement of life quality for cows, herds, and farmers. As such, a principal interest in HHM is to support decisions on how resources best can be transformed into products which the farmer value. In real life situations the HHM professional therefore continuously has to reassess the criteria, which have been used to decide what is 'optimal'. This is possible, if HHM studies are conducted as field studies involving relevant stakeholders since studies 'in the stable(s)' are unarguably complex, dynamic and contextually diverse [4].

Classical experimental research for the evaluation of HHM programs involving herds with or without intervention of some kind may be problematic in applied and highly diverse settings [5]. The reason for this being that both individual farmers and veterinarians will respond to their observations based on previous experiences and own beliefs and in a way that they perceive as optimal [6]. The consequences of such continuous alterations of stakeholders' responses may be reduced data validity associated with the non-exposed herds in the experimental field studies, as these herds cannot be regarded as static controls throughout the study period.

Quantitative field research can be conducted at the individual herd level, which offers some advantages, as modified from Enevoldsen [7]: 1) it becomes possible to conduct within-herd field studies including classical epidemiological techniques; 2) the researcher is close to the collection and processing of data, which makes it possible to relate data and/or results to changes in management; 3) it allows benchmarking or comparison of production results over time to previous measures from the same herd; and 4) is it possible to combine the herd specific context (interviews and dialogue) expressing the farmer's implicit values and goals with 1–3? This complex situation calls for a continuous innovative development of the solely quantitative approach as suggested by Houe *et al. *[8]. This group of authors suggested a more integrated research approach based on the view that epidemiology often integrates different research disciplines. Integrated research may improve the potential for evolving new research fields and increasing validity, precision and transparency of data and results from HHM programs [7]. Others have reported similar considerations [2,4,9]. Integrated research is also a current trend in the (business) management literature [10] and other parts of agricultural economics [11]. Houe and co-authors [8] call for a 'broader approach'. The objectives of this paper are to demonstrate and discuss the need for integrated research within HHM and to introduce the concept of mixed methods research. A practical example of combining different scientific methods will illustrate how uncertainties and biases in databases can be revealed by integrating explicit knowledge giving the background for the included variables.

### Presentation of the quantitative metritis study

In Denmark, there is a legal requirement for all practicing veterinarians to record veterinary treatments (cow identification and diagnosis) whenever drugs regulated by law, e.g. antibiotics or prostaglandins, are administered. All treatments of metritis were therefore assumed to be recorded, and this study aimed at identifying risk factors for cases of metritis treated by a veterinarian. Based on various sources in literature, potential risk factors for metritis were identified, i.e. milk yield, herd size, parity, calving season, breed, other reproductive diseases, digestive disorders, metabolic diseases, nutrition, and age. The objective was to estimate effects of important diseases and other risk factors on the risk of being treated for metritis before 21 days *post partum *utilizing data from the Danish Cattle Database, which stores the treatment records from around 4,600 dairy herds.

The selection criteria for the model were: a) only herds with regular milk control were included; b) only the first registration of a disease diagnosis to be considered a potential risk factor for metritis was included and that registration had to proceed the registration of metritis at cow level or occur the same day; c) the minimum registered incidence rate of disease at herd level was 0.05 treatments per 100 cow years (to reduce the risk of underreporting). Disease registrations from 30 days *ante partum *until 90 days *post partum *from 428,411 calvings occurring from 2003–2005 in 4,647 herds were included in the dataset. Metritis was defined by a single diagnosis (leading to treatment conducted by the veterinarian) recorded between calving and 21 days *post partum*. It was not possible to distinguish between cases having generalized symptoms (i.e. fever) and cases having only local symptoms (i.e. only vaginal discharge) or whether records came from routine fertility programs or the treatment was due to farmers applying for veterinary assistance.

### Presentation of the qualitative interview study on metritis

From a database containing records from routine clinical examinations of fresh cows [7], 71 veterinarians with experience in collecting and processing data according to the 'Danish concept' [12] were identified. In this concept fresh cows are systematically screened, uterine discharge is scored (0–9) and cows are treated for metritis, if they meet the criteria decided in the individual herds. The veterinarians represented 53 different veterinary practices. Twenty veterinarians were randomly selected to participate in a semi-structured interview with the restriction that only one veterinarian per practice could participate in the study. All invited veterinarians accepted the invitation. Interviews were performed by phone and lasted from 10 to 30 minutes and were based on the interview guide in table 1. The interviews focused on the application of criteria for metritis treatment and the metritis scoring system as defined by the 'Danish concept'.

**Table 1:** Interview guide and summaries of the answers of a series of semi-structured interviews.

Questions	Answers from 20 practising veterinarians
1. What percentage of your work is spend working with cattle?	Range from 30–100%
2. How many days *post partum *do you examine the fresh cow?	Fifteen veterinarians performed the clinical examination between 5–12 days *post partum*. Two veterinarians used 4–12 days and three veterinarians used 5–19 days *post partum*.
3. How do you examine the uterus of the fresh cow?	Sixteen veterinarians used vaginal exploration by hand. Two veterinarians only used rectal exploration. Two veterinarians used both vaginal and rectal exploration.
4. Which criteria do you include to diagnose metritis?	Nineteen veterinarians used a standardized metritis scoring system. The last veterinarian used his own scoring system. The nineteen veterinarians used metritis score 5 as a threshold for medical treatment. Three veterinarians consequently used temperature as a diagnostic indicator. Ten veterinarians included temperature on indication (depression, anorexia). Seven veterinarians never used the thermometer. One of these veterinarians explained that he could feel the temperature of the cow during the examination procedure. Another veterinarian told that elevated temperature was indicative to medical treatment.
5. Do you treat all cows according to the same criteria or could there be some considerations that would call for initiation of treatment nonetheless?	Nineteen veterinarians claimed to initiate treatment on identical and repeatable criteria with metritis score 5 as the threshold for medical treatment. However, during the interviews ten veterinarians in retrospect realized that various cow and herd factors (e.g. ketosis, mastitis, reduced milk production, change in behaviour as reported by the farmer, knowledge on metritis problems in the herd or knowledge on a difficult calving) changed their treatment threshold with a range of metritis scores from 4 (three veterinarians); 6 (six veterinarians) and 7 (one veterinarian) for treatment to be initiated.
6. Do you use the score system differently with increasing days in milk?	Four veterinarians said they would treat more aggressively by lowering the threshold for treatment as a consequence of increasing DIM.
7. Do you evaluate the results of the treatments, i.e. control the effect of the treatments?	Nine veterinarians reported that a systematic control effort was unnecessary because of the high success rate in metritis treatment. Nine veterinarians consequently controlled all cows treated at the last visit. Two veterinarians performed controls if the farmer requested it.
8. If you are called to a cow having a badly smelling placenta retained for 4–5 days, how do you then register the case in the Danish Cattle Database?	Twelve veterinarians would register a retained placenta into the database. Two veterinarians motivated this by the price difference between metritis and retained placenta (+ 25%) and that the registrations are combined with the billing system. One veterinarian explained that it was time-consuming to register two diagnoses, so he would only register the retained placenta. Six veterinarians would register a metritis.

### Results from the quantitative metritis study

Approximately 10 percent of the herds had an incidence risk above 18 percent, with a maximum at 39 percent. This indicated that the distribution of the herd incidence risks was highly skewed. The following risk factors were identified in the final model as significantly associated with metritis: Energy corrected milk, herd size, parity, breed, assisted calving, stillbirth, twins, retained placenta, vaginitis, prolapsed uterus, milk fever, ketosis, displaced abomasum, indigestion, traumatic reticuloperitonitis, foot disorder and diarrhoea. Details regarding the results are provided in [13], and will not be further discussed in this paper.

### Results from the qualitative interview study on metritis

The results of the semi-structured qualitative research interviews are summarized in table 1. Important issues were:

• Scoring system. Nineteen veterinarians used the metritis scoring system described above. One veterinarian used his own scoring system despite the presence of very explicit guidelines.

• Time of clinical examination (defined in the manual in the interval 5–12 days *post partum*): Fifteen veterinarians performed the clinical examination between 5–12 days *post partum*. Two veterinarians examined 4–12 days *post partum *and three veterinarians 5–19 days *post partum*.

• Exploration method (not defined in the manual). Sixteen veterinarians used vaginal exploration by hand; two used rectal exploration and two veterinarians used both vaginal and rectal exploration.

• Body temperature (not a parameter included in the manual). Three veterinarians consistently included temperature as a diagnostic tool. Ten veterinarians included temperature on indication (e.g. depression or anorexia). Seven veterinarians never used the thermometer; however; one of these veterinarians explained that he believed he could feel the temperature of the cow during the examination procedures.

• Threshold for treatment. One veterinarian stated that elevated temperature would always lead to a medical treatment. Nineteen veterinarians used metritis score 5 as an indicator of clinical metritis and thus indicative of medical treatment. During the interviews ten veterinarians retrospectively realized that various cow and herd factors (e.g. ketosis, mastitis, reduced milk production, changes in cow behaviour as reported by the farmer, knowledge on metritis problems in the herd or knowledge on a difficult calving) changed their treatment threshold from 5 to one of the following: 4 (three veterinarians); 6 (six veterinarians) and 7 (one veterinarian) for treatment to be initiated.

• Data processing. Twelve veterinarians would record a smelly placenta not expelled 4–5 days *post partum *as 'retained placenta' in the Danish Cattle Database. Two veterinarians were motivated by the price difference (treatment costs) between a case of metritis and a case of retained placenta (+ 25%) to record it as the latter, and charge for this. One veterinarian explained that it was time-consuming to enter two diagnoses into the database, so he would only record the retained placenta. The remaining six veterinarians normally recorded these findings as a metritis.

## Discussion and Evaluation

The relations between risk factors (i.e. retained placenta, parity, milk yield etc.) and metritis in the quantitative research project supported the findings in several other observational quantitative studies [14-16]. However, the results of the semi-structured qualitative interviews already point to potential biases regarding data collection and analyzing data both in purposive sampling and sampling related to routine screenings.

• The veterinarian may examine the cow more carefully if called to attend a "sick" cow.

• The risk of many diseases is higher in early lactation. Consequently, it is likely that more than one disease can be diagnosed. Potential statistical associations may not reflect a biological association between diseases but rather between e.g. lactation stage and disease detection, and therefore reflect bias due to human decision making.

• A veterinarian may initiate medical treatment on basis of an observed predisposing factor such as retained placenta, without actually observing the disease in focus, as indicated in the semi-structured qualitative interview study.

These types of cases cannot be identified in analyses of large databases like the Danish Cattle Database, where medical treatments are recorded irrespective of the farmers' and veterinarians' motivation for treatment and recording. The associations may reflect not only biological relations but also be heavily influenced by decisions taken by the farmer or the veterinarian. The interview results furthermore indicated the presence of herd specific decision making, because most veterinarians included local conditions connected to cow, herd and farmer factors in their decision process. This raises the important issue about what data included in an observational quantitative study actually represents, and it might point to the suggestion that 'the general population of dairy herds' may consist of widely different herds, all subject to individual decision making in their own context. It also points to the important difference between data collected in situations where the farmers and veterinarians focus on a disease outcome and the related risk factors and data collected more 'passively' in relation to disease treatments in different contexts where there is no specific focus on the particular disease. The interview study shows that none of the situations will create uniform data, because perceptions and disease treatment decisions are related to the involved persons. Bartlett and co-authors [17] address this by stating that there is a high variability between veterinarians' diagnostic ability and there is often lack of standardized case definitions. The qualitative research project strongly supports this, and vividly illustrates existing discrepancies in data related to screening of risk animals, also in cases where detailed manuals are expected to standardize the procedures in order to increase comparability of collection and processing of disease data. However, the implicit and individual differences between veterinarians when collecting and processing data may potentially create difficulties when interpreting and inferring across herds. One possible way to handle these contexts related differences would be to perform within-herd experiments, as argued by Enevoldsen [7].

### Skewed Herd Incidence Risk

The highly skewed herd incidence risk of metritis based on treatment data is an important finding. Similar skewed distributions were found in studies of clinical mastitis [17,18]. This 'problem' was handled statistically by selecting a distribution that fitted the data [18] or by cutting off the extreme values due to suspected non-compliance "based on the subjective opinion of the investigators' during the data collection phase" [17]. In the quantitative study described here, herds with very low incidence risks were also excluded. However, there may be simple practical reasons for the skewed distribution like underreporting in many herds [17,19] or significant differences in veterinarians' beliefs in the use of diagnostic tools and in thresholds for treatment, i.e. misclassification errors, as shown in the qualitative research project presented above.

The interview study indicated that an unknown proportion of herds in the database were subject to the veterinarians' more or less systematic clinical examinations, because some herds participated in the described extended herd health program and other herds did not. Thus, cows may have been selected for metritis treatment because of the presence of one or more fixed criteria (e.g. smelling discharge) or known occurrence of expected predisposing risk factors. Consequently, at least 2 types of metritis might be represented in the data utilized in the quantitative study; cases that are truly new incidents and cases that are more or less chronic (or subclinical) because they basically are sampled in a cross-sectional protocol. It may be complicated to distinguish between the 2 types of data based on the available information from the Danish Cattle Database.

### The Methodological Framework of Mixed Methods Research

Qualitative research methodologies and natural scientific research methods do not have any obvious common philosophical or methodological platform. Generally, qualitative approaches are received with scepticism by the natural scientific community because of an accused subjective nature and the absence of 'facts' [11]. However, in many cases the two approaches are mixed. For example, qualitative research results often include some kind of quasi-statistics to report conclusions [10]. Similarly, much quantitative research includes some kind of literature review that is subsequently modified by (qualitative) expert opinion and value(s) [17]. In other words: any researcher will use his or her background and position to judge and select focus areas for an angle of investigations, appropriate methodologies to answer the research question, and interpreting the results and the framing and communication of scientific claims [20,21]. Consequently, contemporary theory of knowledge acknowledges the effect of a researcher's position and perspectives, and disputes the belief of an unprejudiced observer [22].

**Figure 1:**
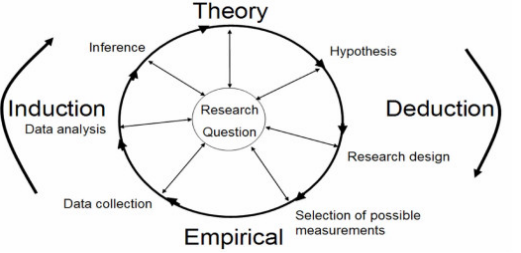
**Conceptual model of the iterative process of induction and deduction in Herd Health Management**. Modified from [19] with inspiration from [2,31].

Mixed Methods Research (MMR) is defined as an intellectual and practical synthesis based on the combination of qualitative and quantitative research methodologies and results [23]. It recognizes the importance of both quantitative and qualitative research methods but also offers a powerful third mixed research methodology that potentially will provide the most informative, complete, balanced, and useful research results. MMR aims at linking theory and practice [19,24] as illustrated in Figure 1. We believe that an appropriate and well-reflected integration of different scientific methods may contribute significantly to the understanding of any data potentially influenced by human action. In the following, it is suggested that scientists with a need to understand a certain field of human action and the consequences and background of these actions can reach far by implementing different methods in their research, and we point to three different methodologies [10,25]: a) supplementary validation; b) triangulation and c) knowledge generation.

### Supplementary Validation

An important use of MMR is to expand primarily qualitative (in particular) or quantitative studies by including other types of scientific methods and data in order to improve and justify a broader understanding of the nature of the results of the studies. Understanding the occurrence of metritis may benefit greatly by supplementary validation, as clearly illustrated by the examples given above. This approach seems to be neglected in most HHM-related publications. This might be understandable, because quantitative observational studies often do not include primary data collection of both qualitative and quantitative nature. Consequently, supplementary validation may often be regarded as 'extra work', i.e. visiting farms again. This paper is an example of supplementary validation.

### Triangulation

The classical definition of triangulation requires that identical findings are reported from separate studies, preferably through different scientific methods [26,27]. Research projects using multiple methods for the purpose of triangulation are characterised by the following two factors: a) the emphasis on testing the same hypothesis multiple times, using different methods in each iteration and b) the focus on aggregating knowledge, rather than on discovering new relationships. That is, each component of a triangulation research project independently illustrates the central argument of the research [25]. The relationship between the component studies is one of joint reinforcement; each component can stand alone, but make a stronger argument in combination. Essentially, this is what happens in the 'Discussion' section of most manuscripts when the (experimental) results are (qualitatively) compared by the authors to previous results reported in literature.

### Knowledge Generation

Herd health management is characterized by an iterative process of refinement of concepts and propositions [2,19]. The initial inductive approach to formulate questions is typical for the iterative process of HHM. Next, the inductive and deductive analyses are mixed [25]. When an epidemiological pattern or a theory has been inductively identified from experimental observations, a hypothesis can be deduced and submitted to testing. The aim of this test would be to reject or accept the generated knowledge in this hypothesis. Consequently, the iterative processes provide new research questions and strengthen conclusions related to the involvement of stakeholders. The multiple stages of inquiry aiming at reframing questions, reconstructing instruments, reanalyzing data and refining interpretations and conclusions all form part of this iterative process. With a mixed design, the different methods are combined into a coherent whole making the evaluation of results a synthesis of all the study data and less a report of findings from each method separately. Mixed designs are generative, yield new insight, or redirect research questions [28].

### The Contribution of Mixed Methods Research in Herd Health Management Studies

Mixed Methods Research is called for in HHM studies to incorporate both perceptions of life and values embedded in the individual dairy setting [7]. As such, MMR requires openness to different views, approaches and perspectives in order to avoid creating barriers to new knowledge [11], including profound modes of thinking and valuing [29]. Disciplines like psychology, sociology, economics and marketing may offer new methodological approaches to the scientific field of HHM. These disciplines have long understood that accounting for individual differences is central to understand the stimulus for change, i.e. 'know thy customer' [30]. This does not imply that HMM professionals should be transformed into social scientists or visa versa, but rather that HHM research may be likely to benefit from a broader approach.

Acknowledgement is needed to the fact that no single research methodology can produce results that are universally transferable and directly applicable without adjustments, when applied in a completely different context [17,22]. This study demonstrates the validity of this statement with regard to the discipline of HHM by example. To understand the actions or preferences of stakeholders [9,21] and approach the diversity of farmers, herds and veterinarians in a scientific manner, the option of conducting farm studies, where more aspects of the herd as well as the human decision making, seems obviously relevant. The results from the two studies on metritis jointly points to the fact that data validity remains a constant challenge.

## Conclusion

Studies where associations between specific herd health management routines and disease outcome variables are based purely on quantitative observational studies multi-herd databases may benefit greatly by adding a qualitative perspective to the quantitative approach as illustrated and discussed in this paper. The combined approach requires besides skills and interdisciplinary collaboration also openness, reflection and scepticism from the involved scientists, but the benefits may be extended to various contexts both in advisory service and science. The need to understand the preferences of stakeholders and the diversity between farmers, herds and veterinarians by combining the strengths of quantitative and qualitative methods and to identify promising solutions to ensure data validity in applied settings remains a constant but rewarding challenge.

## Abbreviations

HHM: Herd health management; MMR: Mixed methods research.

## Competing interests

The authors declare that they have no competing interests.

## Authors' contributions

LNJ collected and analysed the data. EK did the literature review, drafted the manuscript and coordinated among authors. DBN, MV and CE made substantial contributions to the conceptual ideas and revision of the manuscript for important intellectual content in detail. All authors read and approved the final manuscript.
